# Enriched Environment Contributes to the Recovery from Neurotoxin-Induced Parkinson’s Disease Pathology

**DOI:** 10.1007/s12035-024-03951-w

**Published:** 2024-02-13

**Authors:** Daphne Alcalá-Zúniga, Erika Espinoza-Torres, Ranjit Kumar Das, Magaly Vargas, Oscar Maldonado, Omar Benavides, Arvind Manojkumar, Roberto de la Garza, Natalia Davila, Isaac Perez, Alejandro Hernandez Martinez, Deepa Roy, Alejandro López-Juárez, Masoud M. Zarei, Kelsey A. Baker, Mario Gil, Hansapani Rodrigo, Gabriel A. de Erausquin, Upal Roy

**Affiliations:** 1 Department of Health and Biomedical Sciences, University of Texas Rio Grande Valley, Brownsville, TX 78520 USA; 2 Department of Psychological Science, University of Texas Rio Grande Valley, Brownsville, TX USA; 3 Institute of Neuroscience, University of Texas Rio Grande Valley School of Medicine, Harlingen, TX USA; 4 School of Mathematical and Statistical Sciences, University of Rio Grande Valley, Edinburg, TX USA; 5 The Glenn Biggs Institute of Alzheimer’s and Neurodegenerative Disorders, Joe and Teresa Long School of Medicine, University of Texas Health San Antonio, San Antonio, TX USA

**Keywords:** Parkinson’s disease, DJ1, Enriched environment, SH-SY5Y, Cell model, Dopamine, The mouse model

## Abstract

Parkinson’s disease (PD) is a neurological disorder that affects dopaminergic neurons. The lack of understanding of the underlying molecular mechanisms of PD pathology makes treating it a challenge. Several pieces of evidence support the protective role of enriched environment (EE) and exercise on dopaminergic neurons. The specific aspect(s) of neuroprotection after exposure to EE have not been identified. Therefore, we have investigated the protective role of EE on dopamine dysregulation and subsequent downregulation of DJ1 protein using in vitro and in vivo models of PD. Our study for the first time demonstrated that DJ1 expression has a direct correlation with dopamine downregulation in PD models and exposure to EE has a significant impact on improving the behavioral changes in PD mice. This research provides evidence that exercise in EE has a positive effect on PD without interfering with the current line of therapy.

## Introduction

Parkinson’s disease (PD) is the second most prominent neurodegenerative disorder affecting over 8.5 million people globally, with a projected increase to over 12 million by 2040 [1, 2]. A study by the American Academy of Neurology indicated that nearly 1 million people in the USA are affected by PD with 60,000 new cases every year as the population continues to age [2, 3]. The pathology of PD involves the loss of dopaminergic neurons within the substantia nigra pars compacta [4]. Currently available treatments for PD do not target either the cause or progression of the disease. Dopamine replacement pharmacotherapies are very effective against motor impairment in the early stages of the illness but largely leave non-motor manifestations unaffected. When motor fluctuations or other mid-to-late complications of treatment arise, invasive treatments such as deep brain stimulation or enteric delivery of levodopa may become an important part of the management [5, 6]. Therefore, there is a constant need to develop an effective treatment that is less invasive and more effective to prevent disease progression.

Several studies supported that scheduled physical activity or exercise can slow down motor impairment and improve the quality of life in patients with PD [7–9]. There is evidence that the addition of exercise in treatment regimens of patients with early PD has helped against motor and cognitive decline and improved the functioning of patients. It is also supported that the benefits of exercise may extend to non-motor symptoms of the disease [10, 11]. Furthermore, some rodent studies suggest decreased oxidative stress as well as increased production of dopamine supporting evidence that exercise may improve neuroprotection in individuals with PD [12]. Growing evidence also suggests that exercise can be neuroprotective and can reduce cerebral inflammation [13, 14]. Beyond physical activity, the complexity of the environment has shown to have an effect on motor impairment in animal models [15, 16] and patients with PD [16]. Differential housing conditions in experimental animals exposed to dopaminergic neuronal toxins reveal that an EE induces trophic factor release and binding, resulting in improved neuronal survival and motor performance [15]. However, it is unclear what components of the enrichment are responsible for its effects on neuronal survival. At least three different components have been proposed that individually or collectively influence exercise-mediated effects on PD pathology, namely (1) exposure to novelty, (2) intensity or amount of physical exercise, and (3) increased social interactions. Published studies have not addressed which component of EE exactly contributed to the physiological improvement in the brain. Therefore, a lack of understanding of the specific environmental components needed to induce neuroprotection and limited knowledge of underlying molecular mechanism(s) limit the translation of environmental enrichment to clinical settings.

Monogenic forms of PD have been associated with mutations in at least five genes (SNCA, PRKN, DJ1, PINK1, and LRRK2) with a sixth identified as the most common risk factor for the disease (GBA) [17]. Among these, protein deglycase DJ1/Parkinson’s disease protein 7 (DJ1/PARK7) mutations have been long associated with familial PD and have been identified as a possible biomarker for early-onset familial autosomal recessive PD. Although little is known about its specific molecular role, there is enough evidence that suggests DJ1 acts as a regulator of oxidative stress and cell survival in neuronal cells [18]. When the DJ1 protein mutates, it can cause autosomal recessive PD. A very distinct factor for the mutation of DJ1 is its oxidation. Individuals that develop these mutations can have clinical symptoms such as rigidity, tremors, and involuntary movements, as well as neurocognitive decline. Notably, it has also been linked to non-motor symptoms in PD patients [19]. DJ1 is expressed in cells that require significant energy, making it highly involved in the process of protection against oxidative stress. Thus, it shows the importance of DJ1 in the pathophysiology of PD and makes it an ideal biomarker [20, 21]. In addition, dopaminergic neuronal loss in the substantia nigra and depletion of dopamine levels represent a hallmark pathology of PD [18, 22]. Neurodegeneration of dopaminergic neurons can be a consequence of oxidative stress [23–25]. Oxidation and post-translational modifications of DJ1 may affect the way it performs regular homeostatic functions [26].

In this study, we aim to evaluate the response of DJ1 in in vitro and in vivo. For both models, rotenone (ROT) was used to induce a PD-like neuronal injury as per previously published data [27, 28]. Earlier PD models have shown ROT administration in mice can induce motor impairment similar to PD, such as bradykinesia and rigidity [29]. In  in vitro, we evaluated ROT-induced neuronal cell death, whereas in in vivo we assessed first, the impact of ROT on DJ1 modulation, and second, the impact of EE exposure on overall motor performance, cell survival, and DJ1 modulation. Overall, to our knowledge, this is the first study indicating the role of EE in improving the PD pathology and subsequent correlation with the DJ1 expression and dopamine (DA) release.

## Materials and Methods

### Materials

#### Tissue Culture

In vitro experiments were done in human neuroblastoma SH-SY5Y cells obtained from ATCC (ATCC #CRL-2266) and cultured in Eagle’s minimum essential medium (EMEM; Cat.# 30-2003) supplemented with 10% fetal bovine serum (FBS) (Cat.# 30-2020) in a T-75 flask and incubated at 37 °C/5% CO_2_ for 72 h.

#### Drugs and Antibodies

ROT was purchased from Enzo Biochem Inc., NY, USA (Cat. # ALX-350-360-G001) and dissolved in CMC as an excipient. DJ1 and GAPDH primers were obtained from Applied Biosystem, NY, USA, respectively (Cat. # 4331182, Hs00994896_g1 for DJ1 and Cat. # PN4453320, Hs99999905). DJ1 antibody (ab18257) was obtained from Abcam Inc., UK. The rest of the chemicals and reagents were obtained from Fisher Scientific, USA.

#### Animals

For in vivo study, 4–6-week-old male Balb/c (Strain #:000651) mice were purchased from the Jackson Laboratory, Bar Harbor, ME, and housed in pathogen-free cages with free access to food and water under a 12-h light/dark cycle. The animal husbandry was done as per the guidelines for the care of laboratory animals approved by the Institutional Animal Care and Use Committee at the University of Texas Rio Grande Valley.

### Procedures

#### Establishment of ROT-induced PD Cell Model

SH-SY5Y cells were cultured and treated with ROT as per previously established protocols [30–32]. Treatment was introduced when cells reached 60% confluency and were exposed for 72 h to different concentrations of ROT (20, 40, 50, 60, 80, 100, or 150 nM), then tested for DJ1 expression and cytotoxicity.

#### Cell Viability Assay (MTS)

Cell cytotoxicity was measured with CellTiter 96® Aqueous One Solution Cell Proliferation Assay (Promega, Cat. #G358C). SH-SY5Y cells were seeded and grown in a 96-well plate to 60% confluency. Then, the cells were treated with different concentrations of ROT (20, 40, 50, 60, 80, 100, 150 nM) and incubated for 72 h. After an initial wash with phosphate buffer saline (PBS), a total of 20 μl of the MTS reagent was introduced into each well containing 100 µl of fresh media and incubated for 1 h at 37 °C/5% CO_2_. Following that, the plate was read using the Synergy HTX Multi-Mode BioTek plate reader (BioTek, Winooski, VT, USA) to record absorbance at a wavelength of 490 nm after 1 h of incubation. Cells without ROT treatment were used as a negative control. All measurements were taken as a mean of six independent experimental values. The net absorbance value was taken as an indicator of cell viability. The cell viability was calculated as sample/control × 100%. ROT concentrations that affected more than 10% loss in cell viability were considered significantly cytotoxic.

#### Reactive Oxygen Species Assay (ROS)

A ROS assay was performed on SH-SY5Y cells. Cells were grown for 48 h on a 96-well plate. Growth media was discarded from the well and the cells were replenished with fresh PBS + 1% FBS media and incubated for 2 h and this media was used for the remaining part of the experiment. After incubation for 2 h, media was removed and 100 μl of 100 μM DCF-DA was introduced and further incubated for 1 h. After incubation, the media was removed and different concentrations of ROT were added to the wells (20, 40, 50, 60, 80, 100, 150 nM). The plate was incubated for 2 h, then read on the Synergy BioTek Synergy HTX microplate reader (excitation 485 nm and emission 528 nm; BioTek, Winooski, VT). Cells treated with catalase (0.1 mg/ml) were used as a negative control and H_2_O_2_ (50 µM) for 2 h was included as a positive control.

#### Flow Cytometry and Permeabilized Labeling of DJ1 Cells

Cells were permeabilized using solutions containing 0.1% Triton X-100 (Sigma). Nonspecific binding was blocked using 5% Donkey serum in PBS + 1% BSA for 30 min at room temperature (RT). Primary polyclonal anti-PARK7 (Abcam, CA, USA; Cat.# ab18257) was added and incubated overnight at 4 °C. Cells were washed and incubated for 1 h with FITC-conjugated affinity-purified donkey anti-rabbit antibody (Jackson ImmunoResearch Laboratories). Cells were washed and mounted using ProLong antifade with DAPI (Molecular Probes). Images were acquired by a laser scanning confocal microscope (Olympus). All conditions including optical sectioning, number of sections, and exposures were identical for a given set of experiments. For flow cytometry study, cells were scraped from the flask and collected into two groups. One set of cells was used only for permeabilized labeling (see above) in suspension and the other set was exclusively used for flow cytometry (BD Accuri C6). FITC-conjugated affinity-purified donkey anti-rabbit antibody was also used.

#### Immunocytochemistry of DJ1 Protein

An immunocytochemistry study was performed on SH-SY5Y cells grown in chamber slides. Cells were washed with 1X PBS and fixed with ice-cold 4% PFA for 10 min at RT. A washing buffer made from PBS + 0.1 Triton X-100 was added and incubated for 5 min to a total of three times. Primary antibody staining buffer (wash buffer + 10% FBS) was added and incubated for 1 h at RT. Slides were washed with the washing buffer three times; then, the secondary antibody staining buffer was added and incubated for 45 min at RT. The slides were washed and incubated for 5 min for three times. The washing buffer was removed and 1X DAPI in the staining buffer was added and incubated for 5 min at RT. Slides were washed and incubated for 5 min for three times. The slide was mounted with fluoromount G and covered with a coverslip. The primary antibody used was the Rabbit Anti-PARK7/DJ1 antibody (ab18257) at a 1/300 dilution. DAPI at 1/1000 and anti-NeuN at 1/1000. Images observed were taken at 63 × .

#### In Vivo PD Model

Balb/c male mice were used and treated with ROT (5 mice/group). The ROT treatment model was set up based on previous publications and based on our study design [33, 34]. The dosing concentration and schedule were modified based on our previous optimization of neurobehavioral and neuropathological manifestation of the PD at doses of 5, 10, 15, and 30 mg/kg (data not shown). The optimized dose of ROT (10 mg/kg) was administered via the intraperitoneal route every alternate day for 10 days. Mice brain tissues were used for DJ1 gene and protein expression analyses. A set of control mice was injected with a vehicle (CMC or carboxymethyl cellulose) and one control set was untreated. The PD-related parameters were measured by protein expression analysis through gene expression of PD markers and immunohistochemistry. Based on this study, an optimum dose of 10 mg/kg was selected for neurobehavioral study and subsequent exposure to the EE.

#### Treatment

ROT stock solution of 10 mg/kg was freshly prepared before injection. Each mouse was weighed every week to adjust the ROT dosage accordingly. For those mice that received ROT as a treatment and CMC as vehicle control, the injection was administered intraperitoneally (IP) on alternate days between the left and right flanks of the mice. Mice were exsanguinated via cardiac perfusion**.** The mice’s brains were harvested, frozen, and sectioned using a microtome. Midbrain sections with substantia nigra were used for the immunohistochemistry study.

#### Establishment of EE Housing

It has been established that a decrease in motor activity and coordination is part of the hallmark pathologies of PD [35]. To assess the possibility of physical activity providing neuroprotective qualities to the brain, an EE was introduced as a variable in the study. The addition of enrichment items to promote physical activity provided an EE and enhanced sensory stimulation on mice within that condition, which allowed for social and physical interactions. The complexity (the number of objects) of the enriched cage (95 cm · 60 cm · 70 cm, two floors) was increased progressively: every 2 days, two objects were added to the environment. Mice that belonged to the EE and ROT groups had their cages equipped with various toys such as ramps, stairs, hooks, and exercise wheels. Seven days after housing animals in the EE, complexity was kept maximum, but the positions of the objects were continuously changed every 2 days. Two mice were housed together with the EE to promote social interactions as per the published protocol by Gil et al. [36]. Free running wheels were provided but individual animal activity was not monitored. The standard condition (control condition) consisted of cages without any EE components. However, the minimum cage enrichment, such as beddings and nesting materials, was provided.

#### Motor Performance Test (Rotarod)

After 3 and 7 days of ROT treatment, motor performance was observed using a rotarod test as per published protocol [15]. Rotarod analyses were performed to test the rodent’s latency to fall in evaluating the effects on motor coordination and performed at the baseline before the first ROT administration (not included in the results), on day 3, and at the end of treatment. Mice were placed on the rotarod (Cat. #47600, Ugo Basile s.r.l., Italy) and sequentially tested at different speeds from 7 to 25 rpm. When mice fell off the rotarod, they were placed back on it for the remaining time of the test. On testing days, each mouse performed one practice trial (data not shown) to enable the mice to habituate to the rotarod. The trial was carried out after 2 h of rest [33].

#### Neurobehavioral Study

To observe the behavior of mice during their nocturnal cycle, infrared red lights were mounted to provide lighting for the camera. These lights were chosen to prevent unnecessary stress as mice are less sensitive to them. Videos were recorded through the tracking software ANY-MAZE (Stoelting Co.) for 30-min intervals. This software enabled the organization of the test groups and the backing up of the recordings obtained. The files were saved and analyzed for scoring through the event-recording software JWatcher (UCLA). Furthermore, a statistical analysis of the data obtained was performed. These scoring identified the behaviors (locomotor, social inactive, social active, active stationary, inactive stationary) of the mice, as well as their frequency and duration throughout the recordings. Data obtained from the scoring was analyzed through GraphPad (Prism).

Olfactory function was also observed under the same settings and used to record the previous behavioral durations and frequencies. The primary focus of this experiment was to observe enrichment and its effect on the learning curve of these mice. The habituating capabilities of the mice were studied through exposure to olfactory stimuli: water, lime, and almond scents. Each stimulus was given three trials of 3 min for a total of 9 min, after the three trials the stimulus was exchanged. Durations and frequencies for the behaviors were scored using Jwatcher (UCLA) with the inclusion of a nose touch stimulus behavior to indicate an interaction between the animal and the stimulus. Data obtained from scoring were analyzed through GraphPad (Prism).

#### DJ1 Gene Expression Analysis of Mouse Brain Samples (PCR)

Total RNA was extracted from mouse brain using RNeasy Mini Kit (QIAGEN, Germany). DeNovix DS-11 Series Spectrophotometer was used to ensure the purity and concentration of the isolated RNA. To quantify the expression levels of the DJ1 gene TaqMan DJ1 primer (Mm00498538_m1) and GAPDH (Mm03302249_g1) were used as an endogenous control for normalization. PCR amplification was done using a Quant Studio 3 Real-Time PCR System (Applied Biosystems, USA). The thermal cycling conditions consisted of an initial denaturation step at 95 °C for 10 min to activate the polymerase, followed by 40 amplification cycles. Each cycle consisted of denaturation at 95 °C for 15 s, annealing at 60 °C for 30 s, and extension at 72 °C for 30 s. Relative quantification of gene expression was performed using the ΔΔCt method, as described in the referenced study [37].

#### Western Blot Analysis of DJ1 Protein from Mouse Brain Treated with ROT and EE

The brain tissues were harvested from four groups of mice at the end of the behavior study. We performed Western blot experiments to investigate the expression levels of DJ1 protein under various treatment conditions. There were four distinct sample groups: control, ROT, control + EE, and ROT + EE, respectively. Tissue lysate was prepared using RIPA buffer supplemented with protease and phosphatase inhibitors (Cell signaling technology #5872). Then, the lysates were centrifuged at 16,000 g for 20 min. The supernatant was collected, and 4 × Laemmli buffer was added. The mixture was boiled for 5 min at 95 °C to ensure protein denaturation. Protein concentrations were measured using the BCA protein assay. Equal amounts of 15 µg protein were loaded into each well and separated by 4–15% Mini-PROTEAN TGX Precast Gel (Bio-Rad #4561086. Proteins were then transferred to a PVDF membrane using the Bio-Rad Turbo Transfer System. The membrane was blocked with 5% non-fat milk in TBS with 0.1% Tween-20 (TBST) for 1 h at RT. After blocking, the membrane was incubated overnight at 4 °C with a primary rabbit anti-DJ1 antibody (ab18257, Abcam) diluted 1:1000 and Beta Actin 1:3000 (#: sc-47778 Santa Cruz biotech) in 5% non-fat dry milk in TBST. Following primary antibody incubation, the membrane was washed three times with TBST and then incubated with a horseradish peroxidase-conjugated goat anti-rabbit secondary antibody for 1 h at RT. The membrane was washed three times with TBST. Protein bands were visualized using an enhanced chemiluminescence (ECL) detection system. The bands were subsequently captured using Alliance Q9 Imaging System. Beta-actin used as an endogenous control to the normalization of protein expression [37].

For quantification, ImageJ software (version1.53t NIH) was used to analyze the Western blot images. A total of three independent experiments were performed, and from each experiment, three representative images were selected for analyses purpose. Statistical analyses were performed using one-way ANOVA followed by Dunnett’s post hoc test to compare the mean differences in DJ1 protein expression levels among the different groups.

#### Dopamine and Metabolite Analysis of Brain Samples Treated with ROT and/or EE

Mouse brain samples from all four groups were harvested and snap frozen in dry ice and stored at − 80 °C until analysis. In this analysis, six target analytes such as DA, 5-HT, homovanillic acid, GABA, glutamate, and 5-HIAA were analyzed through LC/MS. The brain tissues were homogenized, using a tissue dismembrator, in 100–750 μl of 0.1 M TCA, which contained 10–2 M sodium acetate, 10–4 M EDTA, and 10.5% methanol (pH 3.8). Ten microliters of homogenate was used for protein quantification. Samples were spun in a microcentrifuge at 10,000 g for 20 min at 4 °C. The supernatant was removed for LC/MS analysis. Analytes in the supernatant were quantified following derivatization with benzoyl chloride (BZC). The supernatant (5 μl) was then mixed with 10 μL each of 500 mM NaCO_3_ (aq) and 2% BZC in acetonitrile in an LC/MS vial. After 2 min, the reaction was stopped by the addition of 10 μL internal standard solution. LC was performed on a 2.1 × 100 mm, 1.6 mm particle CORTECS Phenyl column (Waters Corporation, Milford, MA, USA) using a Waters Acquity UPLC. Mobile phase A was 0.1% aqueous formic acid and mobile phase B was acetonitrile with 0.1% formic acid using a gradient starting with 99% A going to 99% B over 19 min. MS analysis was performed using a Waters Xevo TQ-XS triple quadrupole tandem mass spectrometer. The source temperature was 150 °C, and the desolvation temperature was 400 °C [38, 39].

#### Immunohistochemistry

The brain tissue was harvested and sectioned that contained the substantia nigra, at − 3.52 mm from bregma based on the Franklin and Paxinos [40] mouse brain atlas, was identified per animal. An immunohistochemistry protocol was performed on brain tissues obtained from a group under each condition: 10 mg ROT and EE, 10 mg ROT, 10 mg CMC as vehicle control, and no treatment as a negative control. The selected midbrain sections were rinsed (12 × 5-min washes) in PBS at RT to remove the cryoprotectant solution and then stored in PBS at 4 °C. Immediately before immunohistochemistry, sections were rinsed (5 × 5-min washes) in PBS at RT, followed by incubation in 1% hydrogen peroxide in PBS with 0.4% Triton X-100 (PBST) for 10 min at RT to reduce endogenous peroxidase activity. Sections were then rinsed (5 × 5-min washes) in PBS at RT and incubated in rabbit anti-TH polyclonal antibody (cat. # P40101-150, Pel-Freez, USA) (1:1000 dilution) in PBST for 2 h at RT and then an additional  24 h at 4 °C. After incubation in primary antibody, sections were rinsed (10 × 5-min washes) in PBS at RT and then incubated in biotinylated anti-rabbit secondary antibody raised in goat (cat. # 111–005-003, Jackson ImmmunoResearch, USA) in PBS for 1 h at RT. Sections were rinsed (10 × 5-min washes) in PBS at RT and then incubated in avidin–biotin complex (ABC Elite Kit, Vector Laboratories, Burlingame, CA, USA) in PBST for 1h at RT. Sections were rinsed in PBS (3 × 5-min washes), followed by 0.175 M sodium acetate (3 × 5-min washes), and then incubated in 3,3′-diaminobenzidine HCL (0.2 mg/ml, Sigma) with H_2_O_2_ in 0.175 M sodium acetate for 10 min at RT, yielding a brown reaction product. Sections were mounted onto gelatin-subbed slides, air-dried for 48 h, dehydrated, and cover slipped. The images of the slides were taken using a Zeiss Axion. Z1 microscope and representative pictures were used to study the differential TH expression in different groups of mice.

ImageJ software was used to quantify immunoreactive cells in the substantia nigra. The area of the counting frame was 1250 µm^2^, and there were typically 1–5 cells per counting frame, although some frames contained 6 or 7. Twelve counting frames were randomly selected throughout the region of interest (i.e., substantia nigra) for both hemispheres, cells were identified and counted using the ImageJ cell counter plugin, and the average number of cells per 0.1 mm^2^ was calculated for the entire substantia nigra (left and right hemispheres) for each animal.

#### Statistical Analysis

For the in vitro study, cell viability, ROS assay, flow cytometry, and immunocytochemistry were performed in multiple replicates and reported as mean ± standard error. For the behavioral analysis, the non-parametric, Kruskal-Wallis, and Wilcoxon rank-sum tests were used to test the significant variations for behavioral durations among the experimental groups, followed by Holm-adjusted pairwise comparisons using the Dunn’s test. Further analyses on behavioral duration and frequency variations were carried out with linear (for behavioral durations) and generalized (for behavioral frequencies) mixed effect models (with Poisson distribution). Linear/generalize mixed effects (LME/GME) models provide a versatile approach to data analysis and are very useful in multiple research fields as they can handle the non-independence in data. In the current study, data has been collected from the same mouse at multiple time points, which causes non-independence in the data. Hence, with LME/GME, the effect of treatment, stimulus, and their interaction can be accurately evaluated while capturing the variations among the subjects. Note that for the nose touch behavior analysis, the square root transformation (after adding one unit to all observations) has been applied to make the response normally distributed to satisfy the linear mixed effect model assumptions. Power to detect the effects from experimental groups or stimulus in the LME/GME models was assessed by comparing the model specified with the effect, to an alternative model that does not include that effect and was measured with partial ${\eta }^{2}$. The eta squared indicates how much of the total variance in the data is explained by the difference between the means [41]. All tests were two-tailed and performed at a 5% level of significance. Statistical analyses were performed using R statistical software (V.3.6.3) and GraphPad Prism software (GraphPad Prism Software Inc. San Diego, CA).

## Results

### Cell Viability of SH-SY5Y Cells in the Presence of ROT

**Fig. 1:**
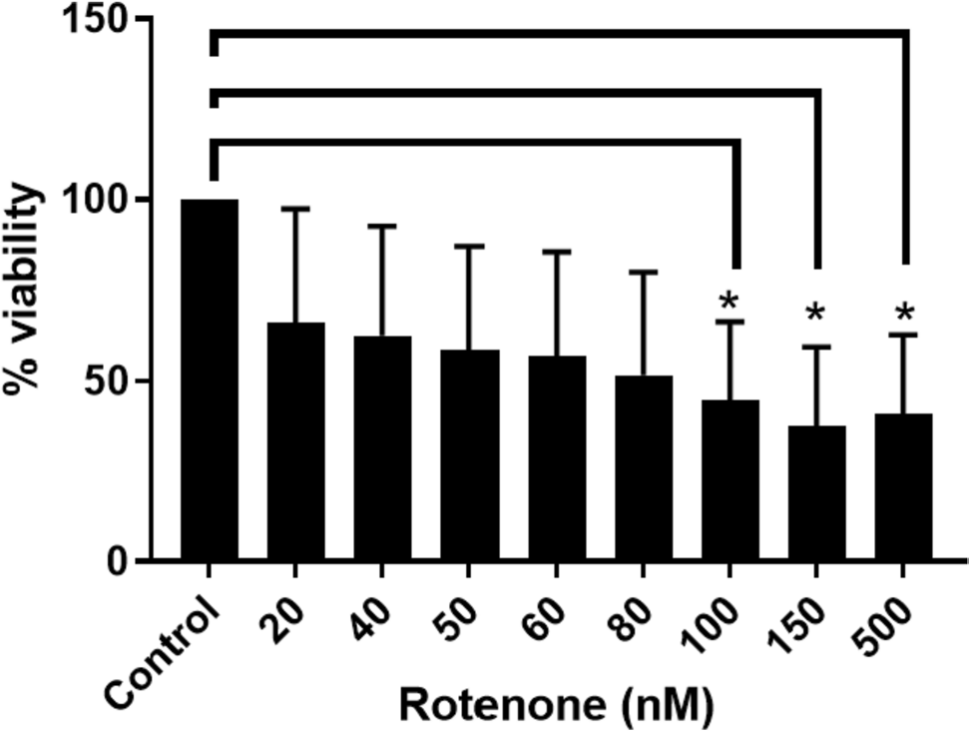
The percentage of cell viability decreases significantly with the addition of the lowest ROT concentration. Neuroblastoma (SH-SY5Y) cells were cultivated in equal environments and then treated with different concentrations of ROT. A cell viability assay (MTS, Promega) was performed and analyzed for absorbance at 490 nm. Cell viability decreases significantly at 20 nm and continues to decrease as ROT concentration increases (*p < 0.05)

The study aimed to assess the expression of the DJ1 protein in the ROT-based PD model. It was important to understand the cytotoxic effect of the ROT on SH-SY5Y cells and determine the concentration that is viable for cell characterization. SH-SY5Y cells were treated with different concentrations of ROT (20, 40, 50, 60, 80, 100, and 150 nM) for an incubation period of 72 h. Results obtained from the MTS cell viability assay suggest that cell cytotoxicity increases as the dosage of the toxin treatment increments (Fig. 1). Analysis through one-way ANOVA reveals a significant difference among the treatment groups with a *p* < 0.0001. The pairwise comparisons between the control and other treatment groups revealed no significant difference (*p*:0.229–0.473). The lowest concentration of 20 nM indicated 60% survival of SH-SY5Y (*p*: 0.473). The highest concentration of 500 nM showed a nearly 50% reduction in cell viability (*p*: 0.019). The concentrations lower than 20 nM and more than 500 nM were not tested based on current published data on in vitro PD model and significant cytotoxicity at higher concentrations respectively [34, 42, 43]. This information also indicated that cells viability was significantly reduced at the higher concentrations of ROT.

### Characterization of ROS Production in the Presence of ROT in SH-SY5Y Cells

**Fig. 2:**
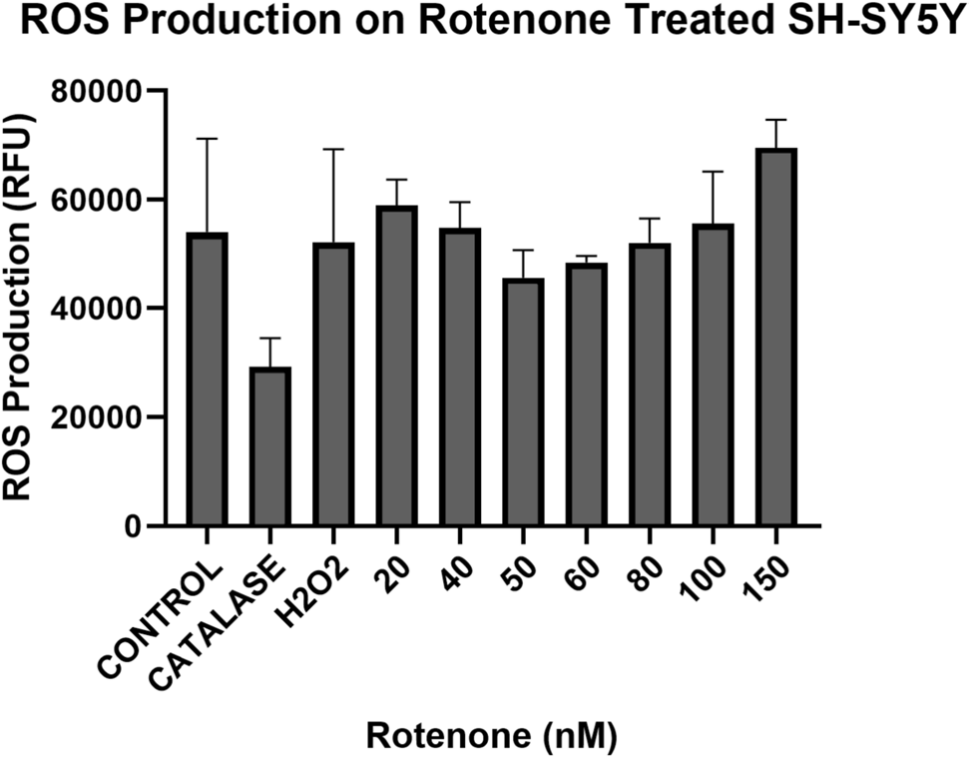
ROS are present when SH-SY5Y neuroblastoma cells are treated with the ROT despite the concentration. An increase in ROS can be observed from the lowest concentration. Quantification of ROS production shows values continue to increase as concentration increases from 60 nM. Analysis through one-way ANOVA reveal a significant difference (*p* < 0.0001)

Data obtained from the ROS assay performed on the SH-SY5Y cells suggest that with the increasing concentration of ROT treatment, the ROS productions were not significantly different (Fig. 2) with a *p:*0.07–0.59. Therefore, with the increasing concentrations of ROT, ROS production was not significantly increased. However, at the concentration of 50–80 nM, there was a decrease in ROS that was not significantly different than any other concentrations (*p*:0.0006–0.0200). Overall, ROS production did not change with the increasing concentrations. Catalase and H_2_O_2_ were used as a negative and positive control respectively.

### Immunocytochemistry and Flow Cytometry of ROT-Treated SH-SY5Y Cells

**Fig. 3:**
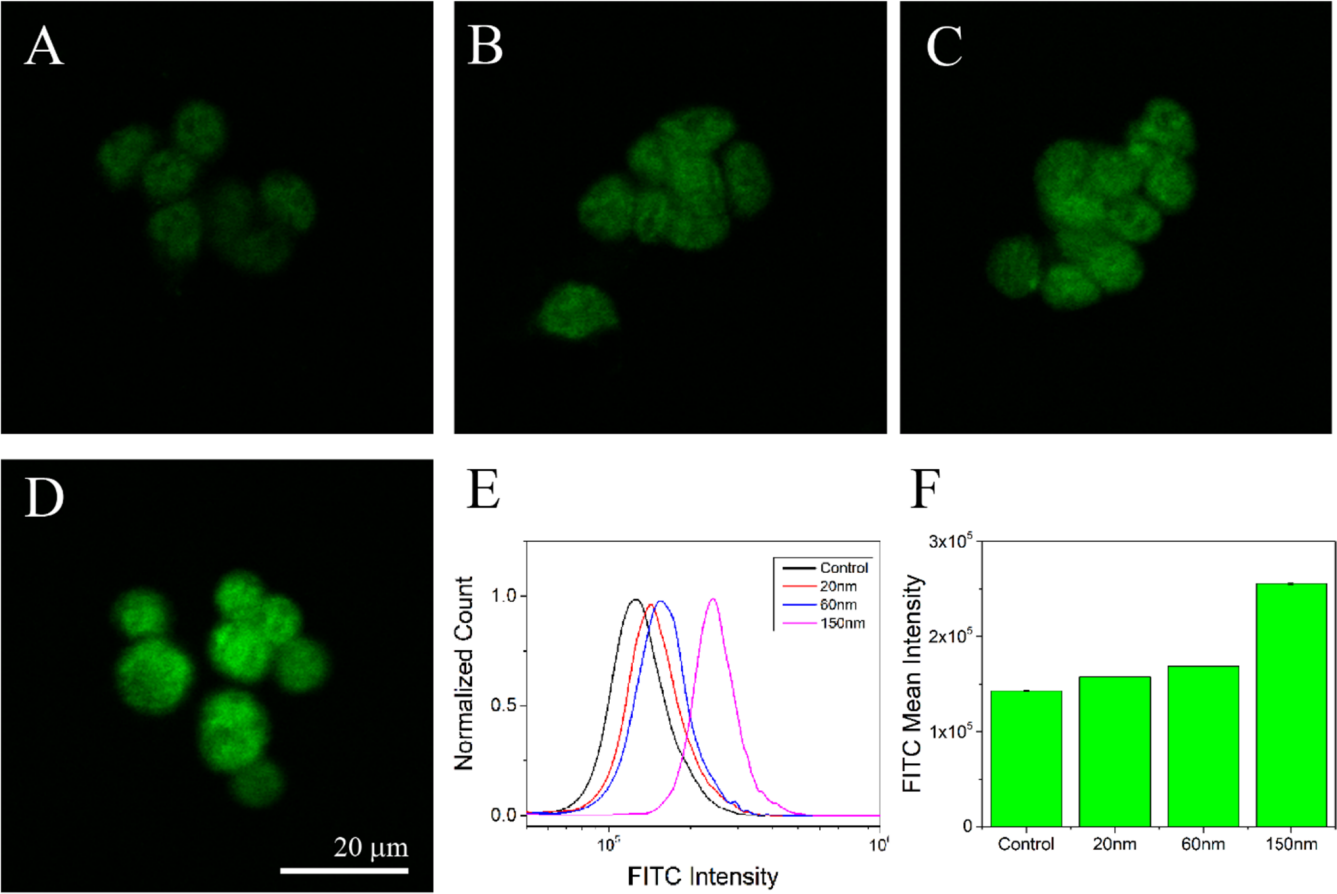
Highest PARK7 expression is observed in cells treated with 150 nm ROT. Neuroblastoma (SH-SY5Y) cells were grown in control (**A**), 20 nm (**B**), 60 nm (**C**), and 150 nm (**D**) ROT for 48 h. Cells were fixed, and labeled with anti-PARK7 antibody (green, FITC). A portion of cells were selected for imaging using a confocal microscope under identical exposure (**A**, **B**, **C**, **D**). The other cell portions were used in flow cytometry experiments (**E**–**F**). Panels **A**, **B**, **C**, and **D** illustrate that by increasing the concentration of ROT from 20 to 150 nm, the PARK7 expression significantly increases. Flow cytometry quantification demonstrates a gradual increase in expression of PARK7 from control to 60 nm ROT and a large jump to 150 nm ROT (**E**–**F**). Normalized count represents 9 to 15 K events. Quantified values are the mean ± SE. Scale bar = 20 µm

Immunocytochemistry of the cell model taken with a confocal imaging system demonstrated upregulation of the expression of DJ1 on the concentrations of 20 nM, 60 nM, and 150 nM of ROT compared to the control (Fig. 3A, B, C, D). The above concentrations were selected considering them being the lowest, medium, and higher concentration since the other concentrations in between did not show significant changes in the cell viability and ROS production. Cell morphology appeared to be affected as the concentration of ROT increased. Cells were tested for DJ1 protein expression through flow cytometry concurrently with immunocytochemistry analysis. The same DJ1 antibody was used to quantitate the DJ1 expression in different ROT treatment conditions. The normalized intensity analysis indicated that there is an upregulation of DJ1 expression compared to the control. However, an increase in the DJ1 expression was not observed to 60 nM. Following that, the 150 nM exposure of ROT significantly increased the DJ1 expression compared to the control. Overall, flow cytometry results indicate that there is a gradual upregulation in the expression of DJ1 protein as the concentration of ROT increases from control to 60 nm, significantly increased at 150 nM (Fig. 3E–F). This observation corroborated with the DJ1 expression in immunocytochemistry.

### Behavioral Study (In Vivo) of ROT and EE-Exposed Mice

**Fig. 4:**
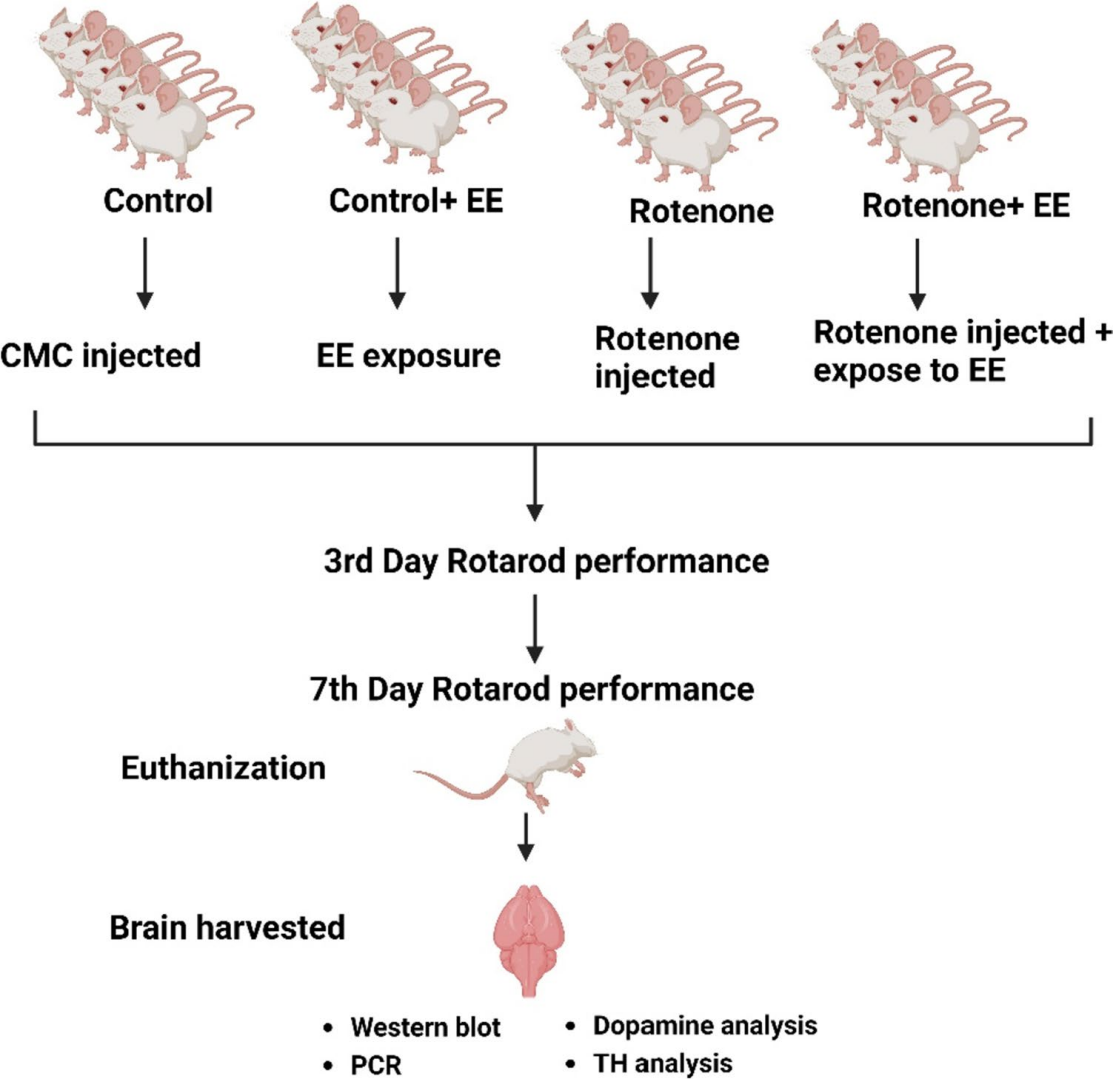
Experimental study design with ROT-treated and EE-exposed mice. The in vivo mice study was involving the establishment of ROT-induced PD model and then exposed them with EE to observe the effects through gene, protein, and behavioral analysis

**Fig. 5:**
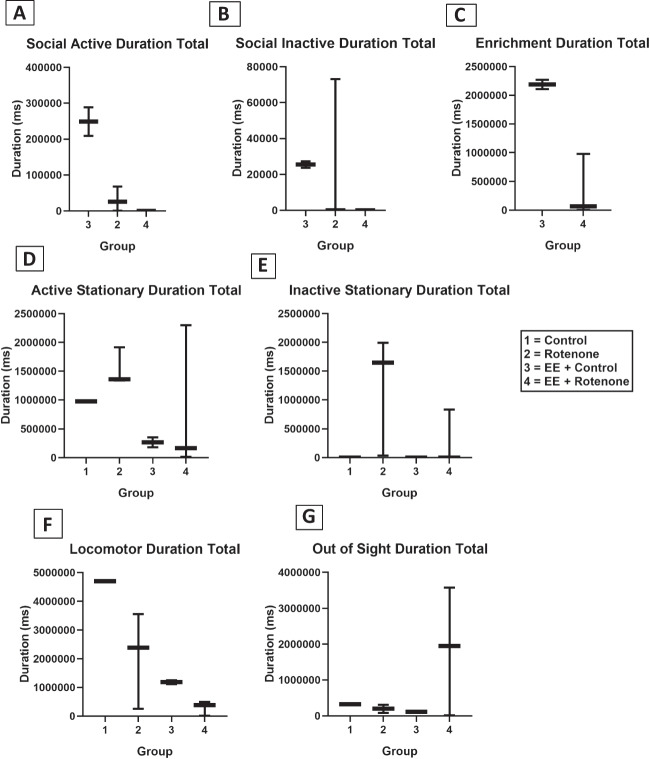
Kruskal-Wallis test revealed no significant changes in the behavioral durations; social active (*p* = 0.59), social inactive (*p* = 0.20), active stationary (*p* = 0.46), inactive stationary (*p* = 0.14), locomotor activity (*p* = 0.21), and out of sight (*p* = 0.63). Enrichment durations (**C**) revealed no significance (*p* = 0.20) (based on the Wilcoxon rank-sum test). Although not statistically significant, there is a noticeable trend in sociability among ROT-treated mice, exerting lower levels of interaction while control animals that received EE had relatively higher levels of interactions (**A**, **B**, **C**). Even within the non-social behaviors (**D**–**E**), there was a trend toward a ROT-induced increase in remaining in one location in the cage (i.e., stationary). There was also a trend indicating that EE may decrease locomotor activity duration in the home cage (**F**). Although control + EE animals spent more time interacting with enrichment items compared to the ROT + EE group, our results confirmed that ROT animals did interact with these items and received stimulation

**Fig. 6:**
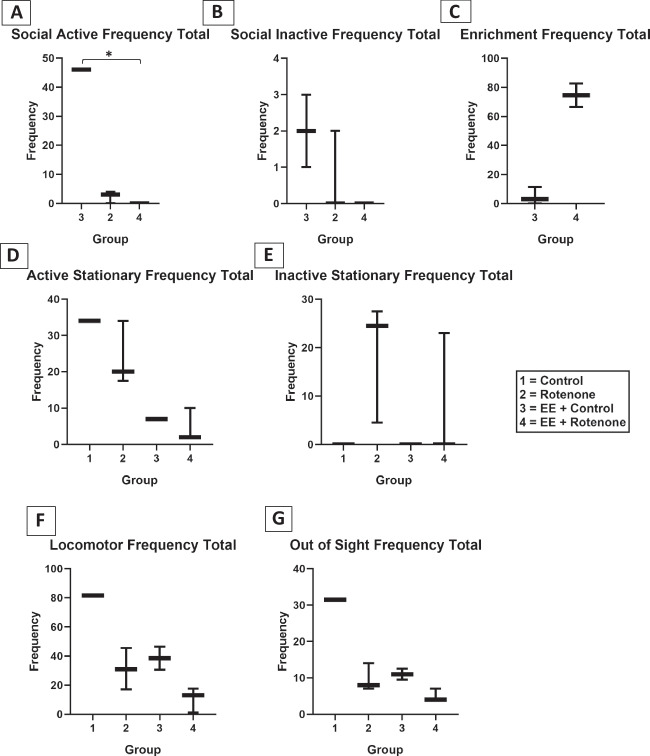
In terms of behavioral frequency, the statistical analyses for each behavior remained identical to those of durations. A significant decrease (*p* = 0.049) in social active behavior from control animals exposed to EE compared to ROT animals exposed to EE. The remaining behaviors displayed no significance: social inactive (*p* = 0.12), active stationary (*p* = 0.092), inactive stationary (*p* = 0.14), locomotor activity (*p* = 0.13), out of sight (*p* = 0.10), and enrichment (*p* = 0.083). Pairwise comparisons among the seven behavioral frequencies with Holm correction revealed no significance between each pair of treatment group. Although no significance, there is an apparent trend in sociability among ROT-treated mice, showing low levels of interaction analogized to the control animals. In terms of non-social behaviors, there was a trend towards ROT-induced increase in remaining in one location in the cage (i.e., stationary). There was also a trend indicating that EE may decrease locomotor activity for ROT-induced groups. Although control + EE animals showed more frequent interaction with enrichment items compared to the ROT + EE group, our results confirmed that ROT animals did interact with EE tools and received stimulation through that

A ROT mouse model was established with the BALB/c mice as per published protocol [33, 34]. Four different groups of mice were included in the study including vehicle (CMC) control mice, mice treated with ROT, mice exposed to EE after being treated with ROT (EE + ROT), and mice exposed to only EE (EE + control), respectively. A dose-response study was designed to observe the effect of ROT in the mice. Three doses of ROT were administered including 10, 30, and 50 mg/kg of ROT for 14 days on alternative days. Based on this observation, 10 mg/kg of ROT was selected for the study, and the study was done for seven days as indicated in the experimental study design (Fig. 4). During a week-long study, the behavioral study was done and at the end of incubation, mice brains were harvested, and the following immunohistochemistry analysis was done. As per Kruskal-Wallis test, the following behavioral duration revealed no significance: social active (H (2) = 5.68, *p* = 0.059), social inactive (H (2) = 3.24, *p* = 0.200), active stationary (H (3) = 2.6, *p* = 0.460), inactive stationary (H (3) = 4.59, *p* = 0.200), locomotor activity (H (3) = 4.56, *p* = 0.210), and out of sight (H (3) = 1.71, *p* = 0.630). Pairwise comparisons among these six behavioral durations with Dunn’s test after Holm correction revealed no significance between each pair of treatment groups (Fig. 5A, B, D, E, F). As per Wilcoxon rank-sum test, enrichment also displayed no significance (*W* = 0.0, *p* = 0.200) (Fig. 5C). Nevertheless, there is a noticeable visual trend in sociability among ROT-treated mice, exerting lower levels of interaction while control animals that received EE had relatively higher levels of interactions (Fig. 5A). In terms of non-social behaviors, there was an apparent trend toward a ROT-induced increase in remaining in one location in the cage (i.e., stationary). Although not statistically significant, our data depicts a trend that EE may decrease locomotor activity duration in the home cage (Fig. 5F). Although control + EE animals spent more time interacting with enrichment items compared to the ROT + EE group, our results confirmed that ROT animals indeed interact with these items and received stimulation. A significant decrease in social active frequencies was found among ROT animals exposed to EE compared to control animals exposed to EE (H (2) = 5.75, *p*:0.049). None of the remaining behavior frequencies were significant: social inactive (H (2) = 4.23, *p*:0.120), active stationary (H (3) = 6.45, *p* = 0.090), inactive stationary (H (3) = 5.55, *p*:0.140), locomotor activity (H (3) = 5.62, *p*:0.13), and out of sight (H (3) = 6.15, *p*:0.100) (Fig. 6A, B, C, D, E, F).

**Fig. 7:**
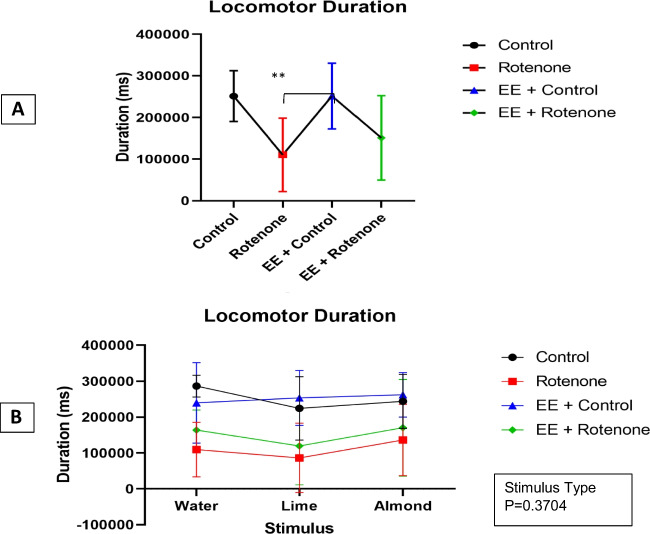
Linear mixed effect (LME) analyses were utilized to evaluate the effect on locomotor behavior durations by the experiment group and stimulus type while considering subjects variations as a random effect. Significant locomotor duration differences were observed among experiment group (*p*-value: < .001), but not with the stimulus type (*p*-value: 0.370) neither their interaction (*p*-value: 0.974).** A** According to the optimal LME model, the expected locomotor duration was 140,850 (ms) [95%CI 46,721.8, 234,978.37] for control group (*p*-value: 0.010), and 141,023 (ms) [95%CI 66,608.0, 215,437.9] for EE + control (*p*-value:0.002) was significantly different from ROT group while the expected locomotor duration for EE + ROT 40,625 (ms) [95%CI − 25,933.9, 107,183.6] was not significant (*p*-value:0.214). **B** The highest mean locomotor behavior durations for all stimulus except water were observed for the EE + control group (water: *M* = 239,450.75, SD = 112,213.27; lime: *M* = 253,109.75, SD = 76,609.75; almond: *M* = 261,776.5, SD = 62,228.02). The lowest mean locomotor behavior durations were recorded for the ROT group (water: *M* = 109,304.5, SD = 75,776.01; lime: *M* = 86,112, SD = 96,687.61; *M* = 135,851.6667, SD = 99,406.48) for all stimulus types. Regardless of the stimulus type the EE + ROT group had shown higher locomotor behavior durations (water: *M* = 163,509.33, SD = 55,755.08; lime: *M* = 119,285.5, SD = 108,600.96; almond: *M* = 170,347.83, SD = 134,765.06) compared to the ROT group

As per the optimal LME model, significant locomotor duration differences were observed with the treatments (${\varvec{\chi}}\left(3\right)=22.78,$ *p*: < 0.001), but not with the stimulus type ((${\varvec{\chi}}\left(2\right)=1.99,$ *p*: 0.370) neither their interaction (${\varvec{\chi}}\left(6\right)=1.25,$ *p*:0.974). A post hoc power analysis reveals 97.5% (95% CI 96.3%, 98.4%) power to detect a large effect size $({\eta }_{p}^{2}=0.85)$ for experimental group with the optimal LME model at a 5% significance level. The expected locomotor duration was 140,850 (ms) [95%CI 46,721.8, 234,978.37] for control group (*p*: 0.010), and 141,023(ms) [95%CI 66,608.0, 215,437.9] for EE + control ($\beta =\mathrm{140,850}, {\text{SE}}=\mathrm{43,002},{\text{df}}=9,$ *p*:0.002) was significantly different from ROT group while the expected locomotor duration for EE + ROT of 40,625 (ms) [95%CI − 25,933.9, 107,183.6] was not significant ($\beta =40625, {\text{SE}}=\mathrm{30,407},{\text{df}}=9,$ *p*:0.214) (Fig. 7A). The highest mean locomotor behavior durations for all stimulus except water were observed for the EE + control group (water: *M* = 239,450.75, SD = 112,213.27; lime: *M* = 253,109.75, SD = 76,609.75; almond: *M* = 261,776.50, SD = 62,228.02) (Fig. 7B). The lowest mean locomotor behavior durations were recorded for the ROT group (water: *M* = 109,304.50, SD = 75,776.01; lime: *M* = 86,112.00, SD = 96,687.61; almond: *M* = 135,851.67, SD = 99,406.48) for all stimulus types. Regardless of the stimulus type the EE + ROT group had shown higher locomotor behavior durations (water: *M* = 163,509.33, SD = 557,55.08; lime: *M* = 119,285.50, SD = 108,600.96; almond: *M* = 170,347.83, SD = 134,765.06) compared to the ROT group.

**Fig. 8:**
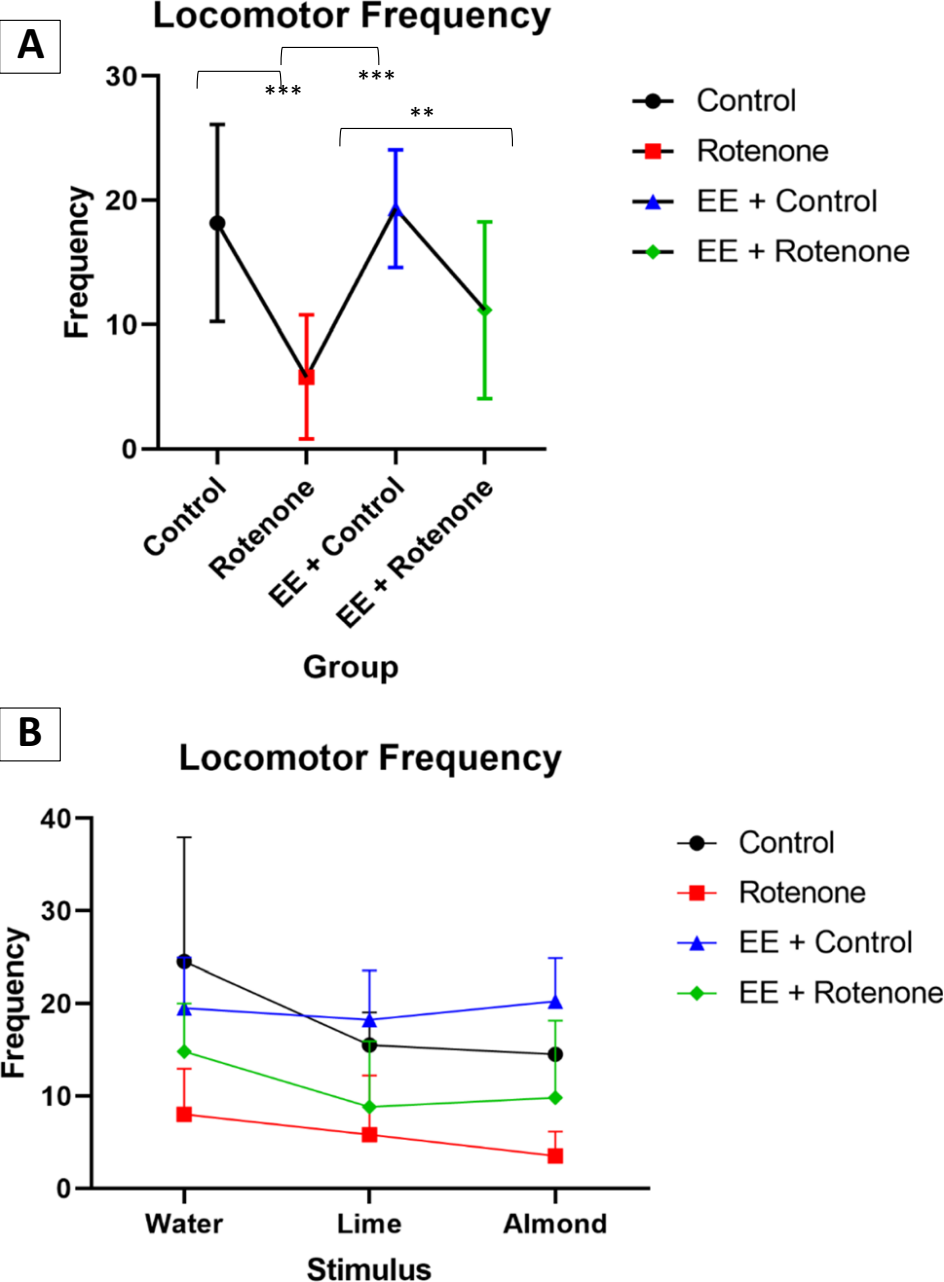
A generalized linear mixed effect (GLME) analyses with Poisson distribution were utilized to evaluate the effect on locomotor behavior frequencies by the experiment group and stimulus type while considering subjects variations as a random effect. Both experiment group (*p*-value: < .001) and stimulus type (*p*-value: < .001) had shown a significant effect on the locomotor behavior frequencies**.** However, their interaction was not significant (*p*-value: 0.064). **A** According to the optimal GLME model, control (*p*-value < 0.001), EE + control (*p*-value < . 001), and EE + ROT (*p*-value = 0.001) had shown significant locomotor frequency differences compared to the ROT group, with an expected locomotor frequency of 3.21 [95%CI 1.78, 5.94] higher for controls, 3.40 [95%CI 2.12, 5.59] higher for EE + control and 1.93 [95%CI 1.24, 3.03] higher for EE + ROT frequencies compared to the subjects within ROT group controlling for the stimulus. **B** The stimulus effect on locomotor frequency for lemon, 0.73 [95%CI 0.60, 0.88] lower compared to water (*p*-value: 0.001), while for almond it was 0.72 [95%CI 0.60, 0.87] with a *p*-value: 0.001. The highest mean locomotor behavior frequencies for all stimulus except water were observed for the EE + control group (water: *M* = 19.5, SD = 5.45; lime: *M* = 18.25, SD = 5.32; almond: *M* = 20.25, SD = 4.65). The lowest mean locomotor behavior frequencies were observed for the ROT group (water: *M* = 8.00, SD = 4.94; lime: *M* = 5.83, SD = 6.37; almond: *M* = 3.50, SD = 2.66) for all stimulus types. Regardless of the stimulus type, EE + ROT group had shown higher locomotor behavior durations (water: *M* = 14.83, SD = 5.19; lime: *M* = 8.83, SD = 7.08; almond: *M* = 9.83, SD = 8.34) compared to the ROT group

In contrast to behavior durations, the treatment (${\varvec{\chi}}\left(3\right)=37.11,$ *p*: < 0001) and stimulus type (${\varvec{\chi}}\left(2\right)=16.42,$ *p*: < 0.001) had shown a significant effect on the locomotor behavior frequencies. However, their interaction was not significant (*p*:0.064). A post hoc power analysis reveals 99.6% (95% CI 98.9%, 99.9%) power to detect a large effect size $({\eta }_{p}^{2}=0.81)$ for experimental group and 95.3% (95% CI 93.8%, 96.5%) power to detect a large effect size $\left({\eta }_{p}^{2}=0.80\right)$ for stimulus type, with the optimal GME model at a 5% significance level. According to the optimal GME model, control ($\beta =1.17, SE=0.27,$ *p* < 0.001), EE + control ($\beta =1.22, SE=0.22,$ *p* < 0. 001), and EE + ROT ($\beta =0.66, {\text{SE}}=0.20,$ *p*:0.001) had shown significant locomotor frequency differences compared to the ROT group, with an expected locomotor frequency of 3.21 [95%CI 1.78, 5.94] higher for controls, 3.40 [95%CI 2.12, 5.59] higher for EE + control and 1.93 [95%CI 1.24, 3.03] higher for EE + ROT frequencies compared to the subjects within ROT group controlling for the stimulus (Fig. 8A). The stimulus effect on locomotor frequency for lemon, 0.73 [95%CI 0.60, 0.88] lower compared to water ($\beta =-0.33, {\text{SE}}=0.09,$ *p*:0.001), while for almond it was 0.72 [95%CI 0.60, 0.87] with a *p*:0.001 ($\beta =-0.32, {\text{SE}}=0.09$). The highest mean locomotor behavior frequencies for all stimuli except water were observed for the EE + control group (water: *M* = 19.5, SD = 5.45; lime: *M* = 18.25, SD = 5.32; almond: *M* = 20.25, SD = 4.65) (Fig. 8B). The lowest mean locomotor behavior frequencies were observed for the ROT group (water: *M* = 8.00, SD = 4.94; lime: *M* = 5.83, SD = 6.37; almond: *M* = 3.50, SD = 2.66) for all stimulus types. Regardless of the stimulus type, EE + ROT group had shown higher locomotor behavior durations (water: *M* = 14.83, SD = 5.19; lime: *M* = 8.83, SD = 7.08; almond: *M* = 9.83, SD = 8.34) compared to the ROT group.

**Fig. 9:**
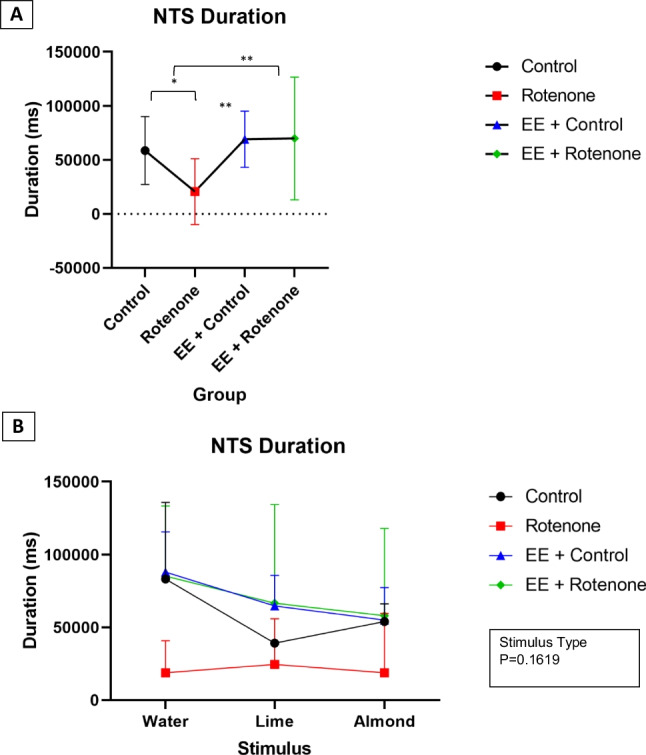
A Linear mixed effect (LME) analyses were utilized to evaluate the effect on NTS behavior durations by the experiment group and Stimulus type while considering subjects variations as a random effect. The square root transformation (after adding one unit for all observations) was applied to make the response normally distributed as to satisfy the LME model assumptions. Only, experiment group (*p*-value: 0.016) effect was significant while stimulus type (*p*-value: 0.616) neither their interaction (*p*-value: 0.950) were significant.** A** According to the optimal LME model, the average NTS duration for the subjects within the control group was 19,078.9 (ms) [95%CI 755.8, 61,880.5] higher compared to the ROT group (*p*-value = 0.023). The average NTS behavior duration for EE + control group was 25,698.3 (ms) [95%CI 5306.1, 61,389.0] higher (*p*-value: 0.003) while this was 19,959.09 (ms) [95%CI 3974.3, 48,179.3] higher for the subjects within the EE + ROT (*p*-value: 0.003) compared to the ROT group. **B** The highest mean NTS behavior durations for all stimulus except water were observed for the EE + ROT group (water: *M* = 85,326.67, SD = 48,229.59; lime: *M* = 66,708.83, SD = 67,854.97; almond: *M* = 58,058.83, SD = 60,057.32). The lowest mean NTS behavior durations were recorded for the ROT group (water: *M* = 18,812.00, SD = 22,000.30; lime: *M* = 24,528.50, SD = 31,426.84; almond: *M* = 18,841.50, SD = 40,737.02) for all stimulus types

**Fig. 10:**
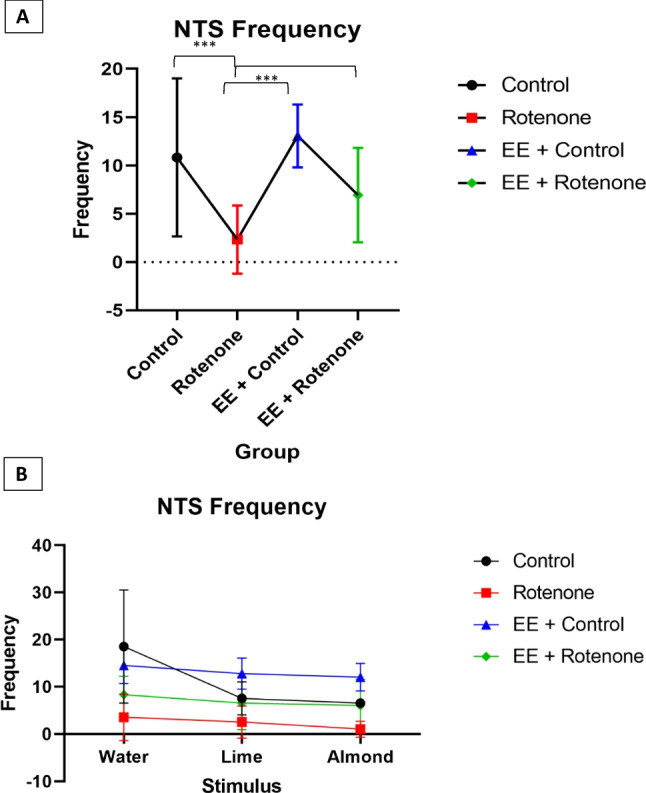
A generalized linear mixed effect (GLME) model with Poisson distribution was utilized to evaluate the effect on NTS behavior frequencies by the experiment group and stimulus type while considering subjects variations as a random effect. Both**,** experiment group (*p*-value: < .001) and stimulus type (*p*-value: < .001) had shown a significant effect on the NTS behavior frequencies**.** However, their interaction was not significant (*p*-value: 0.084). **A** According to the optimal GLME model, subjects within the control group had 4.74 [95%CI 2.04, 11.22] times higher average locomotor behavior frequencies (*p*-value < 0.001), subjects within the EE + control had 5.73 [95%CI 2.91, 11.51] times higher average locomotor behavior frequencies (*p*-value < 0.001), and subjects within the EE + ROT group had 2.80 [95%CI 1.47, 5.25] higher (*p*-value < 0.001) average locomotor behavior frequencies compared to the subjects within ROT group controlling for the stimulus. **B** Similarly, when the subjects were presented with almond and lemon, their average locomotor behavior frequencies were 0.62 [95%CI 0.48, 0.79] and 0.72 [95%CI 0.57, 0.91] lower compared to water with *p*-values: < 0.001 and 0.007, respectively, controlling for the experiment group. The highest mean NTS behavior frequencies for all stimulus except water were observed for the EE + control group (water: *M* = 14.5, SD = 3.87; lime: *M* = 12.75, SD = 3.30; almond: *M* = 12, SD = 2.94). The lowest mean NTS behavior frequencies were observed for the ROT group (water: *M* = 3.5, SD = 4.93; lime: *M* = 2.5, SD = 3.39; almond: *M* = 1, SD = 1.67) for all stimulus types. Regardless of the stimulus type, EE + ROT group had shown higher NTS behavior durations (water: *M* = 8.33, SD = 3.88; lime: *M* = 6.50, SD = 5.65; almond: *M* = 6, SD = 5.55) compared to the ROT group

The effect on nose touch stimulus (NTS) behavior durations with LME revealed a significant effect with the treatments (${\varvec{\chi}}\left(3\right)=10.30,$ *p*:0.016), while stimulus type (${\varvec{\chi}}\left(2\right)=0.97,$ *p*: 0.616) and their interaction (${\varvec{\chi}}\left(6\right)=1.63,$ *p*:0.950) were not significant. A post hoc power analysis reveals 96.6% (95% CI 95.3%, 97.6%) power to detect a large effect size $({\eta }_{p}^{2}=0.72)$ for experimental group with the optimal LME model at a 5% significance level. The average NTS duration for the subjects within the control group was 19,078.9 (ms) [95%CI 755.8, 61,880.5] higher compared to the ROT group ($\beta =138.13,\mathrm{ SE}=50.54,{\text{df}}=9,$ *p*:0.023) (Fig. 9A). The average NTS behavior duration for EE + control group was 25,698.3 (ms) [95%CI 5306.1, 61,389.0] higher ($\beta =160.31,\mathrm{ SE}=39.95,{\text{df}}=9,$ *p*:0.003) while this was 19,959.09 (ms) [95%CI 3974.3, 48,179.3] higher for the subjects within the EE + ROT ($\beta =141.28,\mathrm{ SE}=35.74,{\text{df}}=9,$ *p*:0.003) compared to the ROT group. The highest mean NTS behavior durations for all stimulus except water were observed for the EE + ROT group (water: *M* = 85,326.67, SD = 48,229.59; lime: *M* = 66,708.83, SD = 67,854.97; almond: *M* = 58,058.83, SD 60,057.32) (Fig. 9B). The lowest mean NTS behavior durations were recorded for the ROT group (water: *M* = 18,812.00, SD = 22,000.30; lime: *M* = 24,528.50, SD = 31,426.84; almond: *M* = 18,841.50, SD = 40,737.02) for all stimulus types. Similar to the locomotor behavior frequency analysis, both, the treatments (${\varvec{\chi}}\left(3\right)=34.40,$ *p*: < 0.001) and stimulus type (${\varvec{\chi}}\left(2\right)=16.24,$ *p*: < 0.001) had shown a significant effect on the NTS behavior frequencies. However, their interaction was not significant (${\varvec{\chi}}\left(6\right)=11.15,$ *p*:0.084). A post hoc power analysis reveals 99.8% (95% CI 99.3%, 100.0%) power to detect a large effect size $({\eta }_{p}^{2}=0.79)$ for experimental group and 95.7% (95% CI 94.3%, 96.9%) power to detect a large effect size $\left({\eta }_{p}^{2}=0.80\right)$ for stimulus type with the optimal GME model at a 5% significance level. According to the optimal GME model, subjects within the control group had 4.74 [95%CI 2.04, 11.22] times higher average locomotor behavior frequencies ($\beta =1.56,\mathrm{ SE}=0.38,$ *p* < 0.001), and subjects within the EE + control had 5.73 [95%CI 2.91, 11.51] times higher average locomotor behavior frequencies ($\beta =1.75,\mathrm{ SE}=0.31,$ *p*: < 0.001), and subjects within the EE + ROT group had 2.80 [95%CI 1.47, 5.25] higher ($\beta =1.03,\mathrm{ SE}=0.30,$ *p*: < 0.001) average locomotor behavior frequencies compared to the subjects within ROT group controlling for the stimulus (Fig. 10A). When the subjects were presented with almond and lemon, their average locomotor behavior frequencies were 0.62 [95%CI 0.48, 0.79] and 0.72 [95%CI 0.57, 0.91] which was lower compared to water with *p*: < 0.001 ($\beta =-0.48,\mathrm{ SE}=0.13)$ and 0.007 ($\beta =-0.32,\mathrm{ SE}=0.12$), respectively, controlling for the experiment group. The highest mean NTS behavior frequencies for all stimuli except water were observed for the EE + control group (water: *M* = 14.5, SD = 3.88; lime: *M* = 12.75, SD = 3.30; almond: *M* = 12, SD = 2.94) (Fig. 10B). The lowest mean NTS behavior frequencies were observed for the ROT group (water: *M* = 3.5, SD = 4.93; lime: *M* = 2.5, SD = 3.39; almond: *M* = 1, SD = 1.67) for all stimulus types. Regardless of the stimulus type, the EE + ROT group had shown higher NTS behavior durations (water: *M* = 8.33, SD = 3.88; lime: *M* = 6.5, SD = 5.65; almond: *M* = 6, SD = 5.55) compared to the ROT group.

### Motor Performance Test (Rotarod)

**Fig. 11:**
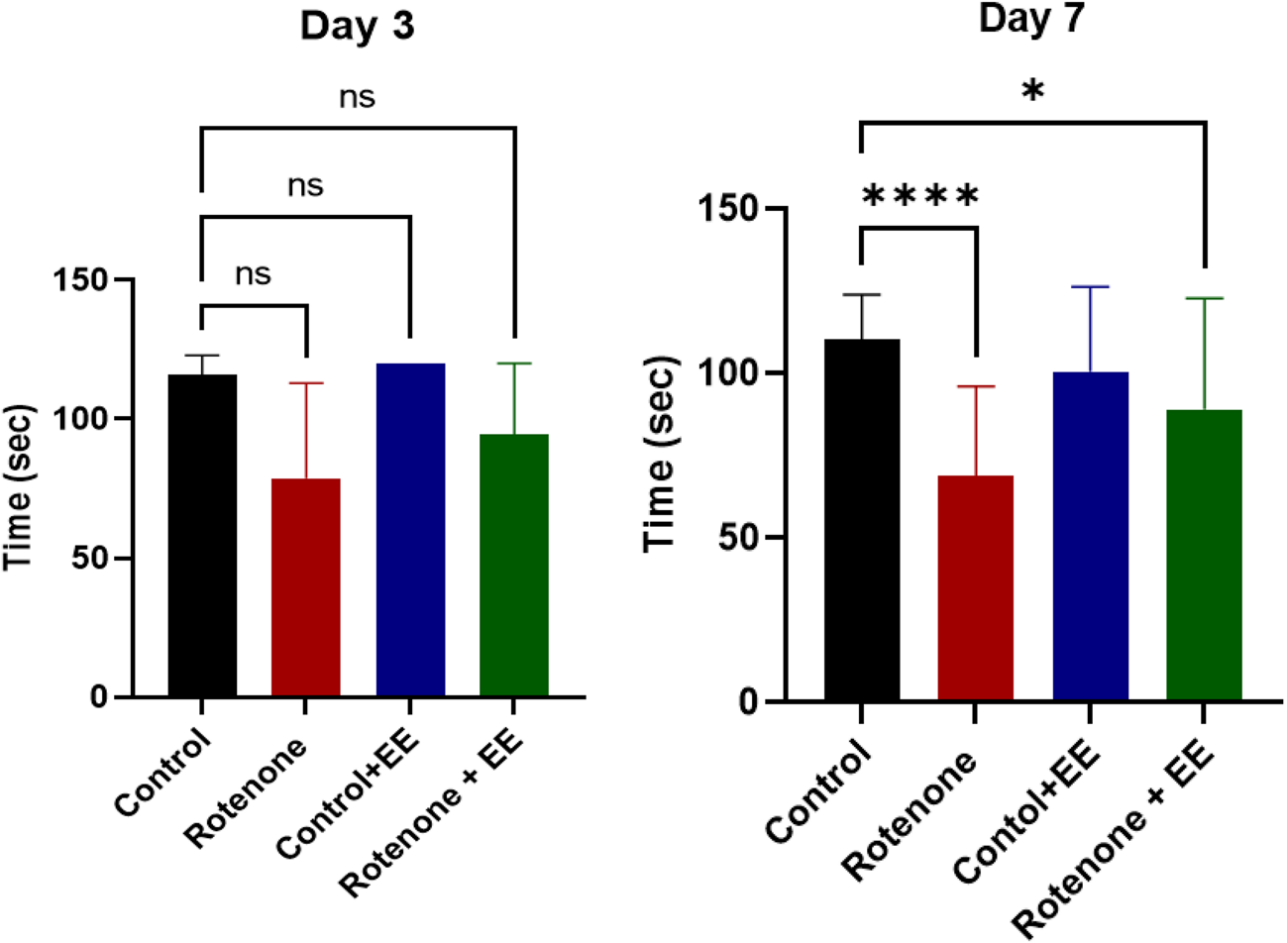
Rotarod performance test for the mice exposed to ROT and EE: Four groups of mice were exposed to ROT and after 3 and 7 days of incubation they underwent rotarod. The mice that only received PBS injection were considered as controls and compared with other treatment groups. The latency to fall off the rotarod was recorded in seconds. Four groups of mice were compared with their latency time and significance analysis was done with the Kruskal-Wallis test. The significance was indicated with p values (*p* < 0.0001)

While the mice were observed under ROT treatment and exposed to EE for 7 days, they were tested for motor coordination test at the end of the study. Detailed analysis indicated that there was no significant difference in motor cognition between control mice and the mice exposed to EE. However, there is a marked difference between the control and ROT-treated groups. Furthermore, statistical analysis between ROT and ROT + EE treated mice showed a significant difference in latency time indicating that ROT-treated mice exposed to EE have better motor coordination compared to only ROT-treated mice as per the post hoc Wilcoxon rank-sum test (Fig. 11, day 7) (*p:* < 0.0001). This observation indicated that EE has a role in preventing the loss of motor coordination in PD pathology.

### DJ1 Gene and Protein Expression in Mouse Brain Treated with ROT and EE

**Fig. 12:**
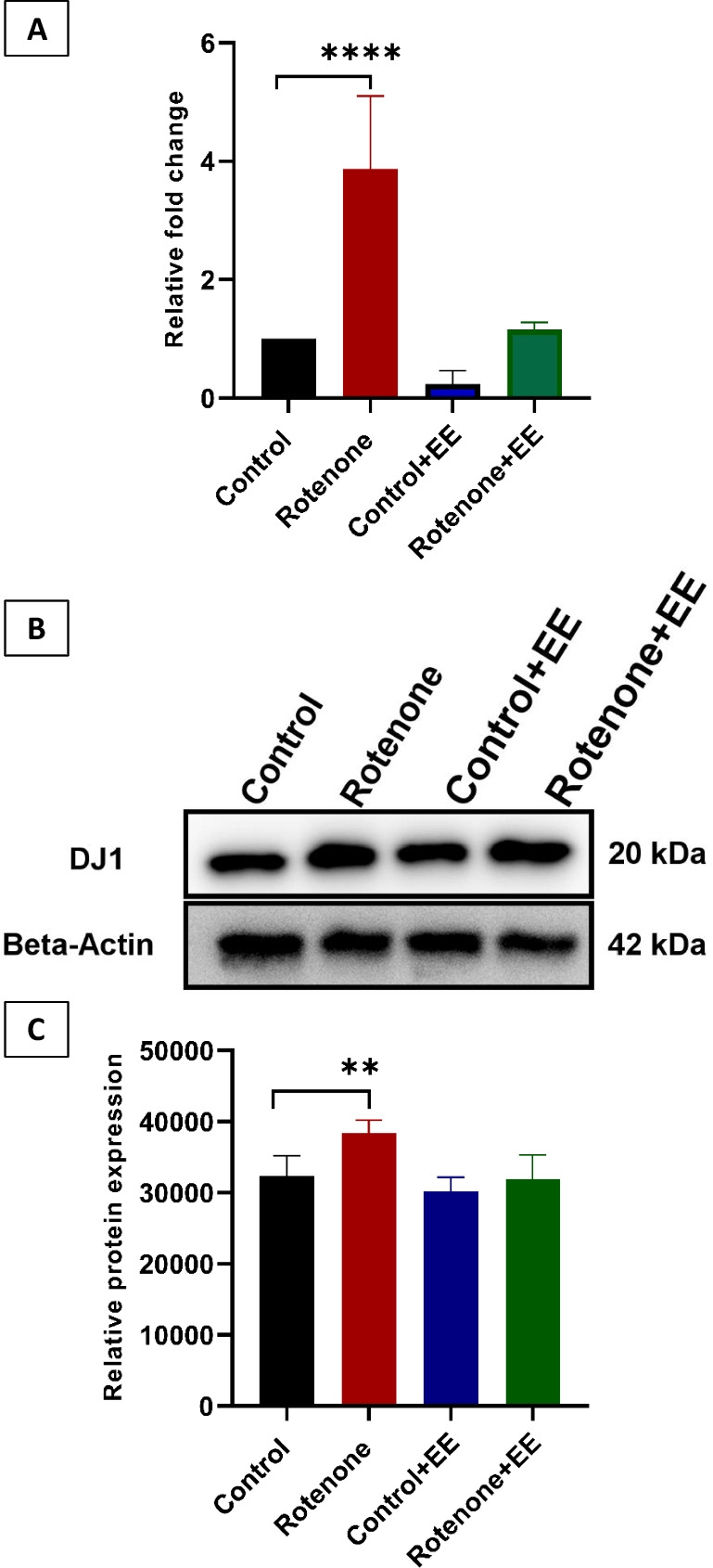
Expression of DJ1 gene and protein is regained on neurons of ROT-treated mice that were exposed to an EE.** A** The mice that were solely treated with ROT showed a significantly higher expression of the DJ1 gene when compared to the control. However, EE treatment had significantly reduced the DJ1 gene expression bringing down similar to control. **B** Western blot data indicated that there was a significant increase in DJ1 protein expression in ROT-treated mice brains. However, the DJ1 protein level remained close to the control mouse brain due to EE exposure. **C** This observation was further confirmed by the volumetric analysis of the multiple protein gel picture through ImageJ analysis. Both gene and protein expression analysis had indicated that there was a significant increase of DJ1 with the ROT exposure which can be normalized through the EE treatment. ****p =  < 0.0001, **p = 0.002

The midbrain section analysis of four groups of mice indicated in DJ1 expression was significantly upregulated in ROT-treated mice compared to control and CMC-treated mice (Fig. 12A). However, DJ1 expression significantly normalized in ROT-treated mice that were exposed to EE. In this regard, it is worth mentioning that the DJ1 expression level of the CMC and EE treated mice (control + EE) is similar to control and CMC-treated mice without any significant difference. As indicated in Fig. 12A, results from PCR analysis suggested the expression of DJ1 gene in substantia nigra is upregulated by ROT treatment. A similar observation was documented with Western blot analysis of DJ1 protein. The DJ1 protein expression analysis indicated that ROT-treated mice had a significant increase in DJ1 protein compared to control. However, control + EE and ROT + EE did not show any significant increase in DJ1 protein expression compared to untreated control (Fig. 12B). This observation was further validated with volumetric analysis of three independent protein gels in ImageJ software (Fig. 12C). The relative protein expression showed the ROT treatment did increase the DJ1 protein expression (*p* = 0.0020). Nonetheless, this overactivation of DJ1 can be normalized by the EE treatment. In contrast, no significant differences in DJ1 expression were observed between the control group and the control + EE or ROT + EE groups. Overall, the gene and protein expression analysis strongly indicated the correlation of DJ1 expression with ROT treatment. In addition, the protective role of EE was also observed in normalizing the DJ1 expression similar to control indicating the recovery of tissue damage caused by the ROT treatment.

### The Effect of ROT Treatment and EE Exposure on DA and Metabolites Synthesis

**Fig. 13:**
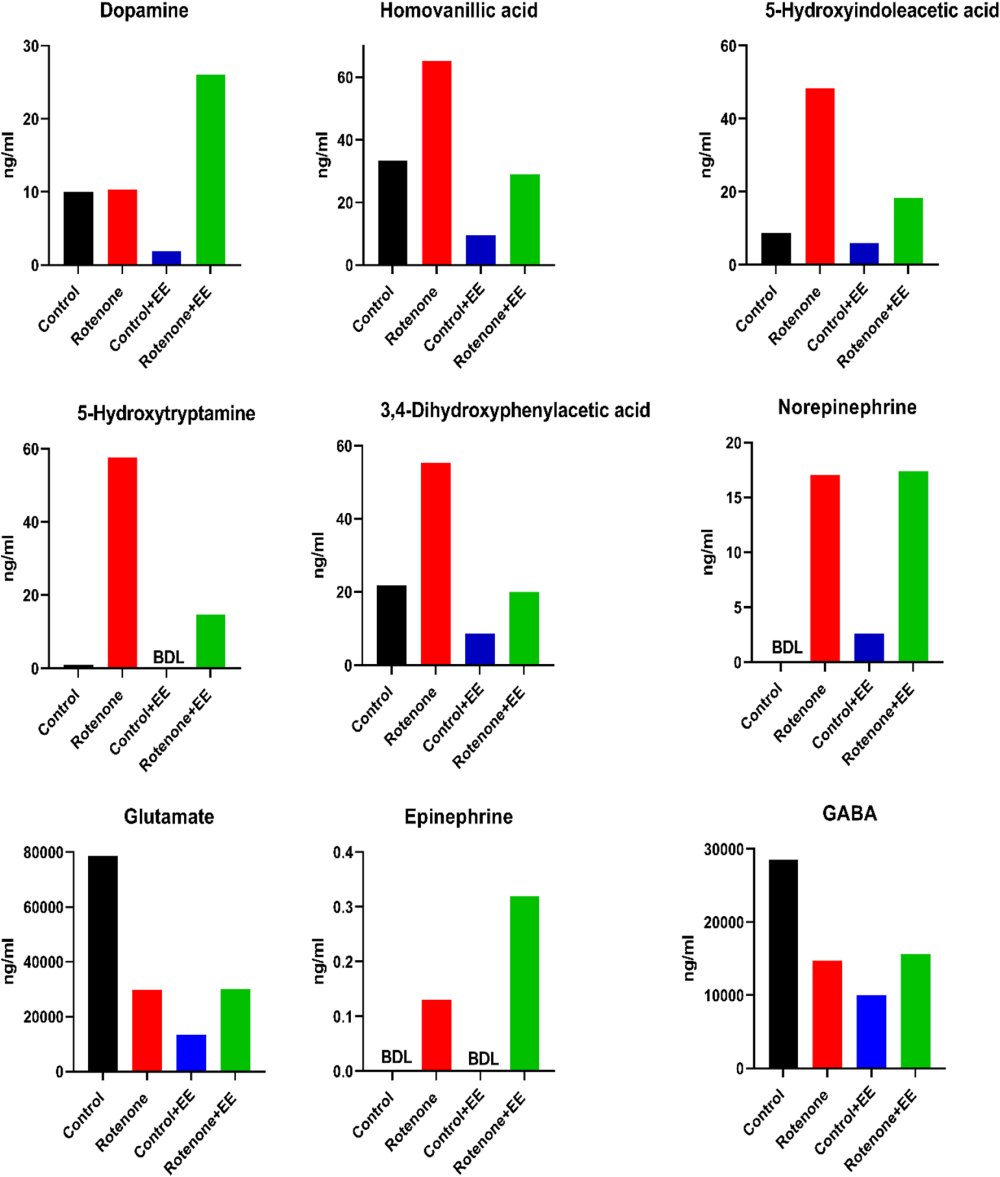
Dysregulation of DA and metabolite synthesis in ROT-treated mice that were exposed to an EE. Mouse brain from four groups (control, ROT, control + EE, ROT + EE) were analyzed for DA, homovanillic acid, 5-hydroxyindoleacetic acid, 5-hydroxytryptamine, 3,4-dihydroxyphenylactic acid, norepinephrine, epinephrine, glutamate, and GABA analysis through LC/MS. The changes in the metabolite are represented in *Y*-axis (ng/ml) and compared with four groups. A noticeable change in DA and metabolite synthesis was observed in ROT-treated mice and ROT-treated and EE-exposed mice

The midbrain tissue sections were dissected, and total protein was extracted for DA, homovanillic acid, 5-hydroxyindoleacetic acid, 5-hydroxytryptamine, 3,4-dihydroxyphenylactic acid, norepinephrine, epinephrine, glutamate, and GABA analysis through LC/MS analysis (Fig. 13). The ROT treatment did not affect DA synthesis. However, homovanillic acid, 5-HIAA, 5-HT, 3,4-dihydroxyphenylactic, and norepinephrine synthesis was induced by the ROT treatment. In addition, there was an increase in synthesis of DA, epinephrine, and norepinephrine in EE-exposed mice treated with ROT. The statistical analysis of the significant change in metabolites could not be performed due to small sample size.

### TH Expression and DJ1 Co-localization in the Midbrain of Mice

**Fig. 14:**
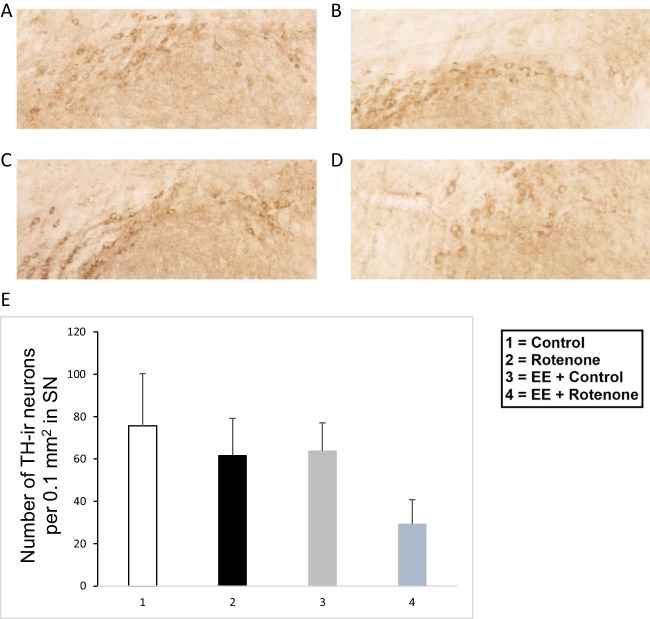
Photomicrographs of TH immunoreactivity in the substantia nigra of selected mice from the four treatment groups: control (**A**), ROT (**B**), EE + control (**C**), and EE + ROT **D**. Although there were no statistically significant differences among groups, the EE + ROT group had on average fewer TH-ir neurons compared to the other groups (**E**)

**Fig. 15:**
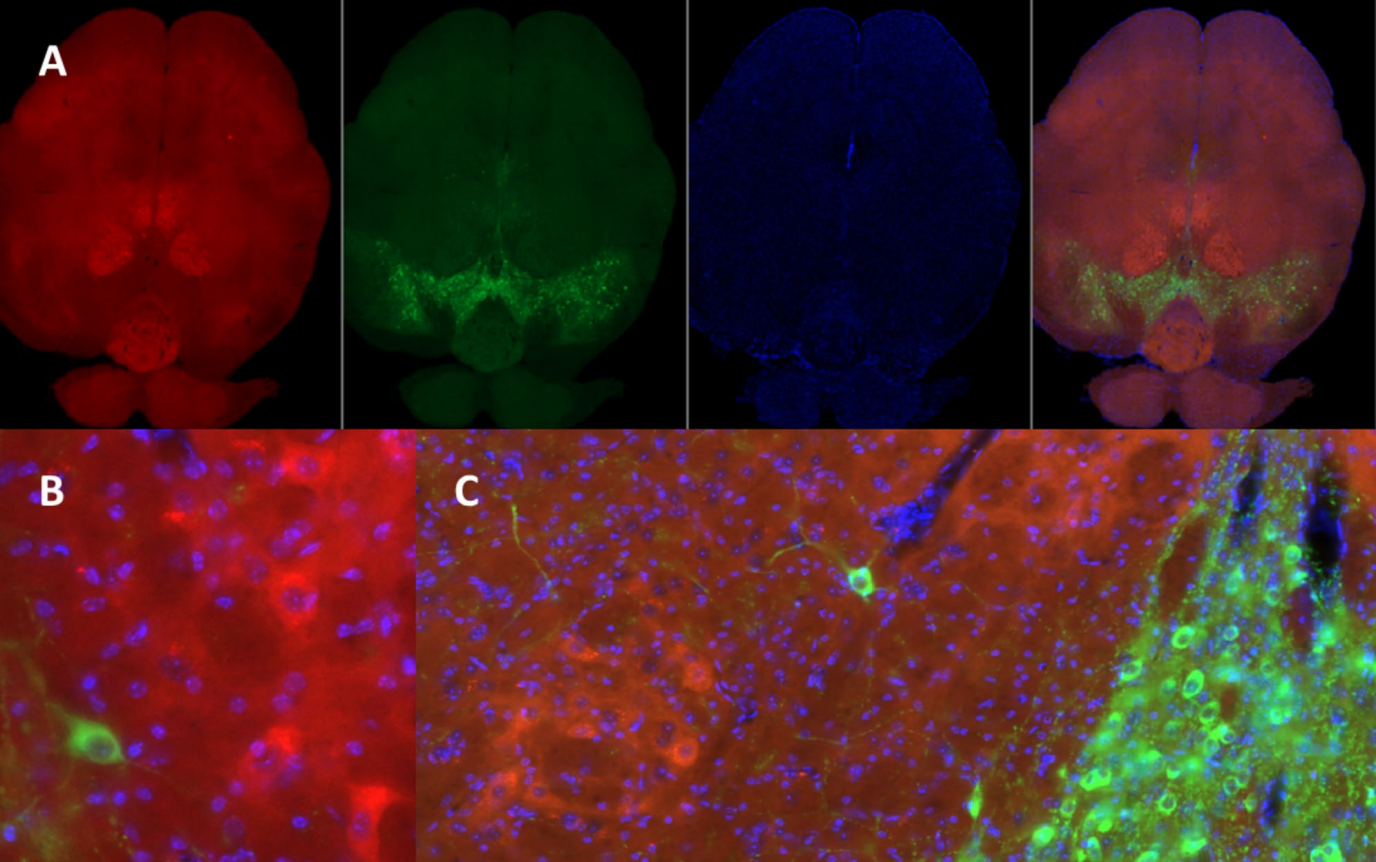
Immunohistochemical localization of TH and DJ1 expression in the ventral midbrain of the mouse. The first panel (**A**) shows that DJ1-ir cells are present in the midbrain (red), TH-ir neurons are, as expected, present in the substantia nigra and ventral tegmental area (green), DAPI counterstain (blue), and all three merged to allow for comparisons. The bottom panel shows that TH-ir neurons are in close proximity to DJ1-ir cells in the midbrain (**B**) and there appears to be a cluster of DJ1-ir cells in the ventral midbrain (**C**), dorsal to TH-ir neurons that comprise the substantia nigra and midbrain

The brain tissue sections that contained the midbrain were also stained using the ABC-DAB method and quantified the number of TH-positive neurons in the substantia nigra. Although there were noticeable group differences (Fig. 14), differences were not statistically significant through Kruskal-Wallis test (*H*(3) = 4.334, *p* = 0.228). However, the control animals and mice that received 10 mg ROT had on average the most TH-positive neurons in the substantia nigra. Interestingly, animals exposed to EE and 10 mg ROT had the lowest number of TH-positive neurons in the substantia nigra. The control brain tissues were further analyzed for co-localization of the DJ1 expression along with TH. It was observed that expression of DJ1 and TH was present in the substantial nigra and ventral tegmental area (Fig. 15A–B). Fluorescent image analysis of these brain sections also indicated that DJ1 and TH-1 are closely localized in two different cells (Fig. 15C). Based on previous analysis (Fig. 3), DJ1 expressions were majorly observed in neuronal cells.

## Discussion

PD is the second most common neurodegenerative disorder that is affecting the aging population across the world [44]. Several studies have characterized that DJ1 mutation can cause monogenic autosomal recessive PD [45, 46]. However, the exact underlying molecular pathway is relatively unknown. Moreover, the multifunctional activity of DJ1 protein and its contribution to overall oxidative stress and neurodegeneration is still relatively unknown [47]. The current study provided a detailed in vitro and in vivo characterization of DJ1 in the ROT-induced PD model. Previous reports have suggested the role of DJ1 in oxidative stress and dopamine regulation [48–50]. The current study has reestablished the DJ1 expression in the PD model confirming the successful establishment of in vitro and in vivo PD models for our study. This study has also provided evidence that EE contributes to the recovery of DJ1 expression and overall behavioral function of the brain in the ROT-induced PD model of mice.

Previous studies have established that characterizations of the neuronal SH-SY5Y cell model treated with ROT suggest that ROT is successful in providing an in vitro model for PD [27, 28, 31]. We confirmed this finding as we observed ROT to have a negative effect on cell viability as indicated in Fig. 1. In addition, the exposure of ROT promoting oxidative stress with increasing concentration was also evident in disease pathology. In this regard, the role is well-known in the clinical setting [51, 52]. However, our study has shown a direct correlation between DJ1 expression and oxidative stress with increasing concentrations of ROT. This data was further confirmed with our immunocytochemistry as well as flow cytometry analysis. The increasing concentration of ROT indicated a higher expression of DJ1 in SH-SY5Y cells. This observation indicated the central role of DJ1 in regulating PD pathology through oxidative stress in in vitro conditions. As it has been established by others that oxidative stress is a key pathology in PD, in the current study the overexpression of DJ1 at the higher concentration of ROT indicated its role in balancing the ROT-induced inflammation in SH-SY5Y cells [53].

Following the in vitro study, the in vivo PD model established the DJ1 expression in the brain tissues that were affected by ROT treatment. The significant difference in DJ1 expression between in vitro and in vivo observation might be due to the level of complexity in the in vivo model and because of the interaction of multiple other factors such as inflammation as well as cell death with a given concentration of ROT. The results of our behavioral studies suggested a complex relationship between EE, ROT, and behavioral outcomes. Not surprisingly, our data indicate that ROT has a negative impact on social interactions, whereas it appears to increase periods of remaining inactive and stationary in one location in the home cage. Although locomotor activity and movement in the home cage are generally considered desirable outcomes, our results suggested that EE has helped in decreasing this behavior in the home cage. One possible explanation for this effect is that in groups where enrichment items are not available, animals may spend more time moving around in the cage, whereas animals that have access to these EE items will spend less time moving around and more time interacting with these items. It should also be noted that we quantified the frequency and amount of time spent interacting with enrichment items, and we found that all animals with access to these items did interact with them. However, there was a noticeable reduction in time spent interacting with EE items, which may suggest that ROT + EE animals received less EE stimulation compared to the control + EE group. The results of the olfactory test demonstrate that ROT inhibits olfactory investigation of neutral odors (almond, lime), which is consistent with other PD models. Remarkably, ROT-treated animals that were housed in EE recovered their ability to investigate odor stimuli (Figs. 8 and 9). Whether this EE-induced recovery in ROT-treated subjects is due to enhanced motivation to interact with environmental stimuli is a question that warrants further investigation.

Among the four groups of mice, the mice treated with ROT indicated upregulation of DJ1 expression and intense PD pathology through gene and protein expression respectively. This observation also reemphasized the role of DJ1 in normalizing the brain pathology that occurred due to ROT exposure [54]. The overexpression of DJ1 in in vitro and in vivo models indicates the increased functional activity of the protein to protect the neurons from dopamine and oxidative stress–induced toxicity [55]. Thus, the normalization of DJ1 expression in ROT and EE-exposed mice ensured the importance of EE exposure during PD pathology. DJ1 expression in untreated and CMC-treated mice indicated a normal level of expression. It is worth mentioning that evidence shows that through EE exposure, present neurons in the substantia nigra are protected from this neurodegeneration by normalizing the DJ1 expression (Fig. 11). When DJ1 fails to be present because of the toxicity induced by ROT, these qualities diminish. The presence of the DJ1 protein in brain samples from mice that were treated with ROT and introduced to an EE is an indicator of its neuroprotective qualities. DJ1 has several different functions including the protection against ROS and DA dysregulation [56]. Thus, the acute overactivation of DJ1 protein in the study indicated the over functioning to bring biochemical balance in the brain tissues that is dysregulated by ROT treatment. This observation has been corroborated with other studies [57, 58]. Nonetheless, it has been established that long-term inhibition or downregulation of DJ1 function was observed in certain PD pathologies due to mutation or other factors [50, 59]. In addition, there was dysregulation of DA and its metabolite in ROT-treated mice indicating the effect of ROT in the brain. This observation also reestablished the DA dysregulation as observed in PD pathology [60, 61] Nonetheless, the study indicated EE-induced synthesis of DA and other metabolites which is supported by other observation in this study. Even though we acknowledge the fact that this observation could not be statistically validated due to small sample size, it provided important information about the role of the EE in restoring the DA and metabolites to recover the PD pathology.

The finding that the EE + ROT group had the lowest number of TH-positive neurons in the substantia nigra was unexpected. However, there are important caveats that should be considered when attempting to interpret these data. First, the impact of ROT on TH-expressing neurons in the midbrain can be variable among animals; that is, as noted by Abdelrazik et al. [62], some animals are particularly sensitive to the toxic effects of ROT on dopamine neurons in the midbrain (and these often become severely ill), whereas for others the impact is less severe. A second point to consider is the time course of toxin-induced effects. In the present study, animals were kept alive long enough for them to complete the behavioral tasks before ending the study. It is possible that a different timeline could yield different results. Given the behavioral data indicating that EE stimulated behaviors in a positive direction, our results suggested that increased TH expression following EE may not be a consistent outcome/marker for EE-associated neuroplasticity in ROT-induced PD models. In addition, our data also suggested that DJ1 is a more reliable and consistent marker in these models. Given that DJ1-expressing cells are present in the midbrain and adjacent to TH neurons (Fig. 12), the role of DJ1 in EE-associated neuroplasticity needs further investigation.

Finally, as noted by Baumans [63], there are several benefits to implementing EE into animal behavior research programs. Major goals of EE include but are not limited to (1) improvement of the quality of life of captive animals, (2) increase in behavioral diversity, (3) reduction in the frequency of abnormal behavior, and (4) enhancement of positive behaviors [49]. A major strength of the present study is that our team incorporated EE into our study design. Our interpretations are based on rich behavioral data that include information about the locomotor function, sensation/perception of stimuli, and social behaviors. Further research is needed to determine whether sustained exposure to EE improves outcomes in classical PD models. Thus, our model has strong translational potential, as it was designed to better reflect the enriching and stimulating environments (and responses to these environments) that are part of the human condition.

## Conclusions

The role of DJ1 has been established in familial autosomal recessive PD. The present study has established the role of DJ1 in oxidative stress and DA dysregulation in the ROT-induced PD in in vitro and in vivo model. In addition, the study for the first time indicated the significant contribution of EE in recovering the general PD pathology through exercise that can be measured through DJ1 expression. Even though exercise is an established tool to countermeasure the progress of PD pathology, the introduction of different components of EE in exercise will significantly increase the motivation and intensity of exercise which eventually improves the PD pathology. In addition, the study raises the possibility that EE may decrease periods of inactivity while stationary in ROT-treated animals, whereas the evidence is stronger that EE increases olfactory investigation in ROT-treated animals. Our data indicated that the relationship among EE, ROT, and social interactions is complex, and more research is needed to determine if social interactions and interacting with the EE tools may play a potential role in PD pathology recovery. The future adaptation of this study can be applied to the clinical setting to improve the PD-related condition along with existing therapy to halt the disease progression.

## Data Availability

All data used in this study is mentioned in the manuscript.
